# Residential trajectories across the life course and their association with cognitive functioning in later life

**DOI:** 10.1038/s41598-022-18501-4

**Published:** 2022-10-11

**Authors:** Dan Orsholits, Stéphane Cullati, Boris Cheval, Paolo Ghisletta, Michel Oris, Jürgen Maurer, Matthias Studer, Adilson Marques, Priscila Marconcin, Élvio R. Gouveia, Matthias Kliegel, Andreas Ihle

**Affiliations:** 1 Centre for the Interdisciplinary Study of Gerontology and Vulnerability, University of Geneva, 1205 Geneva, Switzerland; 2 Swiss National Centre of Competence in Research LIVES – Overcoming Vulnerability: Life Course Perspectives, 1015 Lausanne and 1205 Geneva, Switzerland; 3 Population Health Laboratory, Department of Community Health, University of Fribourg, 1700 Fribourg, Switzerland; 4 Department of Readaptation and Geriatrics, University of Geneva, 1206 Geneva, Switzerland; 5 Swiss Center for Affective Sciences, University of Geneva, 1202 Geneva, Switzerland; 6 Laboratory for the Study of Emotion Elicitation and Expression (E3Lab), Department of Psychology, University of Geneva, 1205 Geneva, Switzerland; 7 Department of Psychology, University of Geneva, 1205 Geneva, Switzerland; 8 Faculty of Psychology, Swiss Distance University Institute, 3900 Brig, Switzerland; 9 Institute of Demography and Socioeconomics, University of Geneva, 1205 Geneva, Switzerland; 10 Department of Economics, Faculty of Business and Economics (HEC), University of Lausanne, 1015 Lausanne, Switzerland; 11 CIPER, Faculty of Human Kinetics, University of Lisbon, 1495-751 Lisbon, Portugal; 12 ISAMB, University of Lisbon, 1649-028 Lisbon, Portugal; 13 KinesioLab, Research Unit in Human Movement Analysis, Piaget Institute, 2805-059 Almada, Portugal; 14 Department of Physical Education and Sport, University of Madeira, 9020-105 Funchal, Portugal; 15 LARSyS, Interactive Technologies Institute, 9020-105 Funchal, Portugal

**Keywords:** Epidemiology, Geriatrics, Public health, Cognitive ageing

## Abstract

Previous work has found that later life urban–rural differences in cognitive health can be largely explained by indicators of cognitive reserve such as education or occupation. However, previous research concentrated on residence in limited, specific, periods. This study offers a detailed investigation on the association between urban (vs. rural) residence from birth, and cognitive functioning in older age. Using data from the Survey of Health Ageing and Retirement in Europe we created residential trajectories from birth to survey enrolment with a combination of sequence and cluster analysis. Using mixed-effects models, we investigated the association between residential trajectories in early, mid, and later life and three cognitive functioning outcomes: immediate recall, delayed recall, and verbal fluency. In a sample of 38,165 participants, we found that, even after accounting for differences related to education and occupation, rural (vs. urban) residence in early life remained associated with poorer cognitive performance later in life. This suggests that growing up in rural regions leads to a long-term disadvantage in cognitive functioning. Thus, public health policies should consider that urban–rural inequalities in early life may have long-lasting associations with inequalities in cognitive health in old and very old age.

## Introduction

Maintaining good cognitive health in older age is crucial for ensuring autonomy and is a key component of active ageing policies^1^. It is thus important to understand which aspects of individuals’ lives are linked to their cognitive health as they age.

The environment in which people live is linked to cognitive functioning in older age with more dense and accessible urban environments being associated with better cognitive functioning^2–4^. The use of more detailed typologies assessing the degree of to which a place of residence is urban or rural in the US, UK, and Ireland has shown that cognitive impairment is more likely to occur in older adults residing in areas far away from major urban areas but also in people living in suburban areas outside of the most densely populated urban regions^5–7^. Moreover, people living in extremely rural environments were more likely to develop cognitive impairment even compared to residents of semi-rural regions^5,8^. Therefore, these findings suggest a clear residential gradient in cognitive functioning as even living in less dense urban areas, compared to highly dense ones, in old age is associated with a greater risk of cognitive impairment.

However, the relationship between cognitive functioning and urban environments is not always consistent and often depends on how individual socioeconomic status is accounted for^9^. Studies have shown that when accounting for the level of education, urban–rural differences in cognitive functioning in older age almost completely disappear especially in younger cohorts^8,10^.

An additional issue raised in the literature is whether the relationship between cognitive functioning in older age and individuals’ residential environment holds when accounting for area of residence in earlier stages of life. While this aspect is comparatively under-investigated, there is evidence from Europe, China, and the United States that people who grew up in rural environments had an overall lower level of cognitive functioning in older age compared to those who grew up in more urban areas regardless of where they lived in old age^11–14^. Yet, these studies only measured early-life or childhood residence at a single point in time corresponding to a specific age (often 14 or 16) rather than across the entire early-life period. This approach provides only a static view of what is a dynamic process and, most importantly, overlooks individuals’ place of residence in the earliest stages of life. Consequently, the link between individuals’ residential trajectories across their whole lives and cognitive functioning in older age, needs to be examined in more depth.

To fill this knowledge gap, this paper investigates the link between individuals’ area of residence in early, mid, and later life, and cognitive functioning in later life using the longitudinal, cross-national Survey of Health, Ageing and Retirement in Europe (SHARE). It goes beyond many approaches investigating the association between early-life rural living and cognitive functioning in older adults by analysing entire trajectories of residence using sequence and cluster analysis rather than focusing on a specific point in time. Using trajectories not only provides a more encompassing view of urban–rural differences, but also takes into account between-individual differences that may have accumulated over people’s entire lives. Moreover, our approach of disaggregating early-life trajectories from those in midlife and during follow-up lets us evaluate the link between area of residence in different life phases and cognitive functioning in later life.

We also assess whether the relationship between region of residence in different life phases and cognitive functioning is attenuated by cognitive reserve. The cognitive reserve concept posits that individual cognitive engagement, often measured by markers such as job skill or level of education^15^ throughout life promotes better cognitive health in older age and has the potential to compensate for neurological problems. There is evidence showing that markers of cognitive reserve are associated with overall differences in cognitive functioning in later life^16,17^. Furthermore, evidence also shows that markers of cognitive reserve attenuate differences in cognitive functioning related to rural vs. urban residence in later life^8,18^. Nevertheless, this relationship has not been investigated systematically in relation to places of residence across the life course. The first aim of this study is to investigate how urban vs. rural residence in different stages of the life course (early, mid, and later life) is associated with cognitive functioning in older age. The second is to assess whether differences in cognitive functioning related to residential trajectories are attenuated when accounting for markers of cognitive reserve.

## Results

Due to the large sample size, we used a cut-off of 0.01 for *p* values.

### Immediate recall

The results from Model 1 for immediate recall (Table 1) showed that respondents living in rural areas, large or small towns, or suburban areas had worse recall scores during follow-up compared to individuals living in big cities even after adjusting for sex, age, health, depression, health behaviours, and country of residence.

**Table 1:** Mixed-effects model estimates for immediate recall.

	Model 1			Model 2			Model 3		
	Est.	95% CI	*p*	Est.	95% CI	*p*	Est.	95% CI	*p*
**Residence during follow-up (ref. big city)**									
Suburbs	− 0.094	[− 0.129, − 0.059]	0.000	− 0.045	[− 0.083, − 0.008]	0.018	− 0.027	[− 0.064, 0.010]	0.152
Large town	− 0.108	[− 0.141, − 0.076]	0.000	− 0.056	[− 0.092, − 0.021]	0.002	− 0.034	[− 0.069, 0.000]	0.053
Small town	− 0.129	[− 0.161, − 0.097]	0.000	− 0.037	[− 0.074, 0.001]	0.055	− 0.003	[− 0.039, 0.034]	0.888
Rural	− 0.169	[− 0.202, − 0.137]	0.000	− 0.020	[− 0.059, 0.020]	0.332	0.016	[− 0.023, 0.054]	0.420
**Early-life residence (ref. big city)**									
Suburbs				− 0.087	[− 0.150, − 0.024]	0.007	− 0.036	[− 0.096, 0.023]	0.230
Large town				− 0.016	[− 0.066, 0.034]	0.527	0.003	[− 0.044, 0.050]	0.902
Small town				− 0.143	[− 0.189, − 0.096]	0.000	− 0.064	[− 0.108, − 0.021]	0.004
Rural				− 0.280	[− 0.322, − 0.238]	0.000	− 0.124	[− 0.163, − 0.084]	0.000
**Midlife residence (ref. big city)**									
Suburbs				− 0.061	[− 0.117, − 0.006]	0.031	− 0.012	[− 0.064, 0.041]	0.663
Large town				− 0.063	[− 0.112, − 0.014]	0.012	− 0.012	[− 0.059, 0.034]	0.605
Small town				− 0.033	[− 0.080, 0.015]	0.184	0.041	[− 0.004, 0.087]	0.076
Rural				− 0.117	[− 0.165, − 0.069]	0.000	0.002	[− 0.043, 0.048]	0.928
**Education (ref. ISCED-3)**									
ISCED-0							− 1.031	[− 1.094, − 0.970]	0.000
ISCED-1							− 0.672	[− 0.706, − 0.637]	0.000
ISCED-2							− 0.315	[− 0.349, − 0.281]	0.000
ISCED-4							0.033	[− 0.026, 0.093]	0.275
ISCED-5							0.311	[0.275, 0.347]	0.000
ISCED-6							0.599	[0.455, 0.742]	0.000
**Job skill (ref. low)**									
High							0.220	[0.191, 0.250]	0.000
Never worked							− 0.326	[− 0.368, − 0.284]	0.000
**Marginal R**^**2**^	0.194			0.199			0.250		
**Conditional R**^**2**^	0.507			0.507			0.509		

*N* = 38,165; *N* observations = 145,593. Adjusted for country of residence, age, sex, self-reported health, depression, and drinking and smoking behaviour. Marginal R^2^ is the variance explained by the fixed effects. Conditional R^2^ is the variance explained by the entire model including the random effects.

*CI*: Confidence interval calculated using likelihood profile method.

Once early- and midlife residential trajectories were included in Model 2, we found that the relative difference between respondents residing in big cities during follow-up and the other residential categories, except large town, became non-significant. The coefficients for residential trajectories in early life showed that individuals who primarily resided in suburbs, small towns, or rural areas remembered 0.09 to 0.180 fewer words from a 10-word list compared to individuals who primarily resided in big cities. For midlife trajectories, we found that only individuals who resided in large towns or rural areas prior to follow-up performed significantly worse compared to those who resided in big cities.

After including the cognitive-reserve indicators (education, and job skill level) in Model 3, we no longer found any statistically significant difference between the different places of residence during follow-up and the different midlife residential trajectories. For early-life residential trajectories, we no longer saw a significant disadvantage for respondents who grew up in suburbs or large towns relative to those who grew up in big cities. However, the negative association between early-life residence in small towns or rural areas remained but was attenuated (0.064 and 0.123 fewer words recalled respectively). For education, we found that respondents with less than an ISCED-3 level of education had lower scores and those with an ISCED-5 or ISCED-6 level of education had higher scores. For job skill level, respondents with highly skilled jobs performed better while respondents who never did paid work had lower scores compared to respondents with low-skill jobs.

### Delayed recall

The results from Model 1 for delayed recall (Table 2) were consistent with those of immediate recall. We again found that individuals who resided outside of big cities during follow-up performed worse on the task.

**Table 2:** Mixed-effects model estimates for delayed recall.

	Model 1			Model 2			Model 3		
	Est.	95% CI	*p*	Est.	95% CI	*p*	Est.	95% CI	*p*
**Residence during follow-up (ref. big city)**									
Suburbs	− 0.145	[− 0.187, − 0.104]	0.000	− 0.089	[− 0.133, − 0.045]	0.000	− 0.070	[− 0.114, − 0.026]	0.002
Large town	− 0.174	[− 0.213, − 0.136]	0.000	− 0.109	[− 0.151, − 0.067]	0.000	− 0.085	[− 0. .127, − 0.044]	0.000
Small town	− 0.188	[− 0.226, − 0.149]	0.000	− 0.071	[− 0.116, − 0.027]	0.002	− 0.035	[− 0.079, 0.009]	0.118
Rural	− 0.232	[− 0.270, − 0.193]	0.000	− 0.040	[− 0.087, 0.006]	0.091	− 0.002	[− 0.048, 0.044]	0.934
**Early-life residence (ref. big city)**									
Suburbs				− 0.095	[− 0.172, − 0.018]	0.016	− 0.038	[− 0.111, 0.036]	0.316
Large town				− 0.006	[− 0.067, 0.055]	0.846	0.016	[− 0.042, 0.074]	0.581
Small town				− 0.172	[− 0.228, − 0.115]	0.000	− 0.085	[− 0.139, − 0.031]	0.002
Rural				− 0.359	[− 0.410, − 0.308]	0.000	− 0.184	[− 0.233, − 0.135]	0.000
**Midlife residence (ref. big city)**									
Suburbs				− 0.055	[− 0.123, 0.013]	0.113	− 0.002	[− 0.067, 0.063]	0.956
Large town				− 0.086	[− 0.146, − 0.026]	0.005	− 0.029	[− 0.086, 0.029]	0.326
Small town				− 0.040	[− 0.098, 0.018]	0.178	0.041	[− 0.015, 0.097]	0.148
Rural				− 0.155	[− 0.213, − 0.097]	0.000	− 0.021	[− 0.077, 0.035]	0.458
**Education (ref. ISCED-3)**									
ISCED-0							− 0.937	[− 1.049, − 0.896]	0.000
ISCED-1							− 0.707	[− 0.749, − 0.664]	0.000
ISCED-2							− 0.373	[− 0.415, − 0.331]	0.000
ISCED-4							0.040	[− 0.034, 0.114]	0.292
ISCED-5							0.371	[0.327, 0.416]	0.000
ISCED-6							0.738	[0.561, 0.916]	0.000
**Job skill (ref. low)**									
High							0.274	[0.238, 0.311]	0.000
Never worked							− 0.304	[− 0.356, − 0.252]	0.000
**Marginal R**^**2**^	0.177			0.183			0.227		
**Conditional R**^**2**^	0.515			0.515			0.517		

N = 38,165; N observations = 146,006. Adjusted for country of residence, age, sex, self-reported health, depression, and drinking and smoking behaviour. Marginal R^2^ is the variance explained by the fixed effects. Conditional R^2^ is the variance explained by the entire model including the random effects.

*CI*: Confidence interval calculated using likelihood profile method.

Once early-life and midlife residential trajectories were included (Model 2), we found that only individuals residing in rural areas during follow-up no longer performed significantly worse compared to those residing in big cities unlike for immediate recall. For early-life residence, in line with the results for immediate recall, we found that respondents who grew up in rural areas or small towns performed significantly worse (0.359 and 0.172 fewer words recalled). For midlife residence, we found that respondents living in rural areas or large towns had lower scores compared to those who lived in a big city.

The inclusion of the cognitive reserve markers in Model 3 further attenuated the association between area of residence during follow-up and delayed recall. Individuals residing in small towns and rural areas no longer performed significantly worse than individuals living in big cities. Additionally, there was no longer any significant difference between areas of residence in midlife. However, for early-life residence, individuals who resided in rural areas or small towns still performed significantly worse than respondents who lived in big cities (0.184 and 0.085 fewer words recalled).

As for the indicators of cognitive reserve, the results followed the same pattern as for immediate recall. Respondents with less than an ISCED-3 level of education had lower scores and those with an ISCED-5 or ISCED-6 level of education had higher scores. For job skill level, respondents who had a high-skill job performed better while respondents who never did paid work had lower scores compared to those with low-skill jobs.

### Verbal fluency

The results from Model 1 for verbal fluency (Table 3) were similar to those for immediate and delayed recall. During follow-up, respondents who did not reside in a big city had lower scores on the verbal fluency test with individuals residing in small towns and rural areas performing the worst.

**Table 3:** Mixed-effects model estimates for verbal fluency.

	Model 1			Model 2			Model 3		
	Est.	95% CI	*p*	Est.	95% CI	*p*	Est.	95% CI	*p*
**Residence during follow-up (ref. big city)**									
Suburbs	− 0.208	[− 0.353, − 0.063]	0.005	− 0.065	[− 0.218, 0.088]	0.403	0.005	[− 0.146, 0.156]	0.948
Large town	− 0.206	[− 0.342, − 0.071]	0.003	0.013	[− 0.133, 0.159]	0.860	0.096	[− 0.048, 0.239]	0.192
Small town	− 0.366	[− 0.501, − 0.231]	0.000	− 0.011	[− 0.166, 0.143]	0.887	0.114	[− 0.038, 0.266]	0.143
Rural	− 0.352	[− 0.489, − 0.216]	0.000	0.140	[− 0.024, 0.303]	0.094	0.282	[0.121, 0.443]	0.001
**Early-life residence (ref. big city)**									
Suburbs				− 0.294	[− 0.572, − 0.016]	0.039	− 0.096	[− 0.360, 0.174]	0.480
Large town				− 0.237	[− 0.457, − 0.018]	0.034	− 0.162	[− 0.373, 0.048]	0.131
Small town				− 0.491	[− 0.696, − 0.285]	0.000	− 0.197	[− 0.394, − 0.000]	0.050
Rural				− 1.008	[− 1.194, − 0.823]	0.000	− 0.406	[− 0.585, − 0.227]	0.000
**Midlife residence (ref. big city)**									
Suburbs				− 0.015	[− 0.257, 0.226]	0.900	0.156	[− 0.077, 0.388]	0.189
Large town				− 0.298	[− 0.512, − 0.084]	0.006	− 0.100	[− 0.305, 0.106]	0.342
Small town				− 0.285	[− 0.493, − 0.078]	0.007	− 0.008	[− 0.208, 0.192]	0.936
Rural				− 0.403	[− 0.610, − 0.196]	0.000	0.058	[− 0.142, 0.257]	0.572
**Education (ref. ISCED-3)**									
ISCED-0							− 3.020	[− 3.295, − 2.745]	0.000
ISCED-1							− 2.224	[− 2.377, − 2.070]	0.000
ISCED-2							− 1.186	[− 1.338, − 1.034]	0.000
ISCED-4							0.537	[0.267, 0.808]	0.000
ISCED-5							1.347	[1.185, 1.508]	0.000
ISCED-6							2.224	[1.576, 2.872]	0.000
**Job skill (ref. low)**									
High							1.087	[0.954, 1.219]	0.000
Never worked							− 1.166	[− 1.353, − 0.978]	0.000
**Marginal R**^**2**^	0.255			0.258			0.298		
**Conditional R**^**2**^	0.613			0.613			0.613		

N = 38,165; N observations = 127,377. Adjusted for country of residence, age, sex, self-reported health, depression, and drinking and smoking behaviour. Marginal R^2^ is the variance explained by the fixed effects. Conditional R^2^ is the variance explained by the entire model including the random effects.

*CI*: Confidence interval calculated using likelihood profile method.

The inclusion of early- and midlife residence in Model 2 attenuated this relationship as there was no longer any statistically significant difference between the different places of residence during follow-up. For early-life trajectories we found similar results to those for recall with respondents who resided in rural areas and small towns having lower scores (1.008 and 0.491 fewer words listed) compared to individuals who resided in a big city. For midlife residence, respondents who lived in large and small towns, or rural areas performed worse compared to those who resided in big cities. However, those who resided in suburbs did not perform worse relative to those who resided in big cities.

Once the cognitive reserve indicators were included in Model 3, for residence during follow-up we found that respondents who lived in a rural area performed better compared to those who resided in big cities and there was no significant difference for the other categories. For early-life residence, only respondents who lived in rural areas, compared to a big city, had significantly lower scores (0.406 fewer animals listed) compared to those who lived in a big city. For midlife residential trajectories, there was no longer any significant difference between the different categories.

For the cognitive reserve indicators, the results were similar to those for immediate and delayed recall. Having a high-skill jobs was associated with higher scores and never having had paid employment was associated with lower scores. For education, the results were also in line with those for the other outcomes, with ISCED levels 0 through 2 being associated with lower scores and ISCED levels 4 through 6 being associated with better scores relative to ISCED level 3.

## Discussion

Our results contribute to explaining the mixed findings reported in the literature concerning the association between the area of residence and cognitive health in older age. Without taking into account early- and midlife residential trajectories or markers of cognitive reserve, we observed that individuals living outside of highly urban areas performed worse on the immediate recall, delayed recall, and verbal fluency tasks. This result is in line with studies that only consider respondents’ area of residence during follow-up. The more detailed typology utilised in SHARE also allowed us to evaluate whether there was a gradient in cognitive performance in relation to how rural an individual’s place of residence was. For all outcomes, the results for Model 1 showed a clear gradient with respondents having progressively lower scores the less urban their place of residence was. This is in line with results previously found in the literature^5–8^.

Nevertheless, the inclusion of early- and midlife residential trajectories in our models made differences between respondents residing in big cities and other areas during follow-up completely non-significant. This result suggests that the urban–rural differences observed in older age can potentially be attributed to some form of cumulative disadvantage related to having grown up in a rural environment. It is consistent with previous work that posits that early-life urban or rural living may better explain differences in older age than contemporaneous urban or rural living^11,12^.

We also found that early-life area of residence remained associated with cognitive functioning in later life even when accounting for education and job skill level (the two markers of cognitive reserve). This was not the case for midlife residence. Overall, our findings are in line with previous research showing that cognitive reserve attenuates differences in cognitive functioning in older adults linked to urban vs. rural residence in old age^8,10,18^.

Consequently, our results suggest that the environment in which individuals lived in early life, but less so in later life stages, plays an important role in their development which in turn can have a long-lasting influence on cognitive functioning in later stages of the life course. This notable result brings support to the critical period hypothesis^19^ and shows the importance of early-life conditions for cognitive health in older age.

One possible explanation for why living rural environments in early life carries a disadvantage in terms of cognitive functioning in later life is that less dense rural environments provide fewer opportunities for cognitive stimulation than more dense urban ones. A more stimulating cognitive environment can be extremely important in childhood as it can lead to better cognitive development through exposure to more multisensory contexts earlier in life^2,20^. Building on this line of thought, the area of residence in early life can also determine the opportunities individuals have to accumulate cognitive reserve, especially among older cohorts such as those in our study, by conditioning access to higher quality education in earlier life stages which is more often available in highly urban areas^21,22^.

As for why mid- and later-life residence were not associated with cognitive functioning when accounting for cognitive reserve, a possible explanation is that individuals who chose to pursue higher education or who were employed in highly skilled jobs gravitated, or were “socially channelled”, towards more urban areas after their early life course stages. In other words, it is likely that later-life urban–rural residential differences could be conflated with educational and occupational differences.

A further contribution of the study is the investigation of how markers of cognitive reserve influence the relationship between cognitive functioning and urban–rural differences in early, mid, and later life simultaneously. Our findings show that education and job skill level completely explain differences in cognitive functioning between urban and rural residence in mid and later life for immediate recall and verbal fluency. They also reduce differences in cognitive functioning associated with residential trajectories in early life, with the reduction being strongest for individuals who lived in rural areas. Education and occupation provide cognitive stimulation later on in life^8,15^, which can potentially serve to counteract the disadvantages related to less cognitive stimulation and lower quality education associated with living in rural areas early on in life. Thus, the disadvantage of rural living in early life can still be reduced through cognitive stimulation in later stages of the life course.

Despite strengths such as a large cross-national sample, the use of longitudinal data, and the analysis of residential trajectories across the entire life course rather than at specific ages, there are nevertheless limitations. The first is related to how the area of residence is determined. In SHARE, the area of residence is a subjective rather than objective measure, and it is assessed retrospectively for life course trajectories. This means that the classification of place of residence is not necessarily established using the same criteria in all countries. However, this is also the case for typologies based on objective indicators which also vary across countries and studies^23^. Nevertheless, the question is harmonized across all countries and waves, and the impact of recall bias in SHARE for retrospective data is low^24^. In addition, the area of residence during follow-up was evaluated by the interviewer rather than the respondents, the categories (big city, suburbs, large town, small town, and rural area) are consistent across all waves, and they are the same for both the retrospective assessments and interviewer evaluations.

The second limitation is that the current study is observational and therefore cannot establish any causal relationship between place of residence and cognitive functioning in older age. Nevertheless, the criterion of temporal precedence is met in the case of early- and midlife residential trajectories as they refer to life phases that occurred before the cognitive functioning evaluations done during the survey.

Taken together, our results show that cognitive functioning in later life is associated with individuals’ place of residence during their early life course but not during the middle or later stages. The association between early-life rural residence and cognitive functioning also remains significant when controlling for markers of cognitive reserve. Thus, our results suggest that there is a disadvantage associated with growing up in rural areas, which has a long-lasting impact on cognitive functioning that can only be partially attenuated by cognitive reserve accumulated through education or work. To promote better cognitive ageing outcomes in later life, public health policies should encourage individuals from rural areas to pursue higher education in earlier life stages as this can reduce the disadvantage of growing up in rural areas for cognitive health in later life and lead to better ageing outcomes.

There are also opportunities for future research. One direction that looks promising is to further leverage the multi-country nature of SHARE to explore how the relationship between place of residence over the life course and cognitive functioning varies. It is likely that differences in social institutions, policies, geography, and other country-specific characteristics can affect how disadvantageous rural residence in different life stages can be for cognitive functioning in later life. Therefore, certain countries may be better at limiting the long-term disadvantage we observed for early-life rural residence than others. This could serve to illustrate potential changes that could be made to improve cognitive functioning in old age for individuals who spend the early parts of their lives in rural regions.

## Methods

### Participants and sample

We used data from the Survey of Health, Ageing and Retirement in Europe (SHARE). SHARE is a biennial panel survey of individuals aged 50 years and older and their partners, in Europe and Israel^25^. The first wave of data collection occurred in 2004–2005, with latest available interviews dating from 2019–2021 (wave eight). In the third and seventh waves, SHARE collected retrospective life course data using a life-history calendar as part of the SHARELIFE module^25^.

The sample used for this study included individuals aged between 50 and 90 (mean 69.7, SD 8.7) living in 19 European countries (Austria, Belgium, Czech Republic, Denmark, Estonia, France, Germany, Greece, Hungary, Ireland, Italy, Luxembourg, the Netherlands, Poland, Portugal, Slovenia, Spain, Sweden, and Switzerland) and Israel. We selected these countries because they all conducted the life-history calendar module at least once.

Individuals who were *not classified* as retired during the retrospective survey waves were also excluded. Dropping further observations with missing information on any item used in the analysis (complete case analysis) resulted in a final sample of 38,165 respondents.

The SHARE was approved by the Ethics Council of the Max Planck Society and the relevant research ethics committees in the participating countries which confirmed that the study was conducted in accordance with the Declaration of Helsinki and all relevant legal and ethical regulations. All participants in the SHARE provided written informed consent.

### Outcomes

We analysed three indicators of cognitive functioning: immediate recall, delayed recall, and verbal fluency. For immediate recall, the interviewer read out a list of 10 words and respondents had one minute to list as many words as they could recall immediately after hearing them. For delayed recall, respondents had to list the same words as for the immediate recall task in the same amount of time after having completed additional cognitive functioning evaluations. For verbal fluency, respondents were given 1 min to name as many animals as possible. Descriptive statistics for these variables and additional information on how the tests were conducted in the SHARE are available in the Supplementary Appendix. The full cognitive functioning module was conducted in all waves except waves three (no cognitive functioning evaluations) and seven (no verbal fluency test).

### Independent variables

Area of residence over the life course was measured using three variables derived from subjective evaluations of the area a place of residence was located. The first was the area of residence during follow-up which was evaluated by interviewers at each wave for each household. The area of residence was assigned one of five categories by the interviewer: a big city, the suburbs or outskirts of a big city, a large town, a small town, or a rural area.

For early-life and midlife residence we used retrospective data on respondents’ places of residence since birth. Each respondent was asked once, in either wave three or wave seven, to list all their places of residence. For each place of residence, the interviewer asked the respondent how they would describe the area of residence using one of five categories: a big city, the suburbs or outskirts of a big city, a large town, a small town, or a rural area.

**Figure 1:**
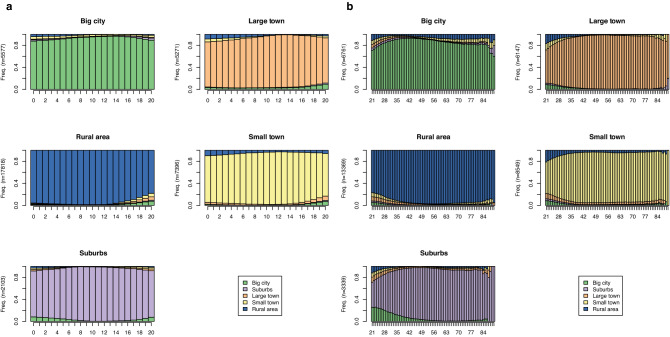
Sequence density plots for the five clusters of early-life residential trajectories (**a**) and midlife residential trajectories (**b**). For each cluster, the bars represent the proportion of respondents living in one of the five residential areas at a specific age.

Using this information, we constructed sequences using the TraMineR package^26^ charting individuals’ areas of residence from birth until age 20 for early-life and then from age 21 until they joined SHARE for midlife. Respondents with incomplete information or gaps in their trajectories until study entry were excluded. We then used a spell-length optimised version of optimal matching (OM) with constant substitution costs^27^, in combination with hierarchical clustering using Ward’s method to create a typology of early-life and midlife residential trajectories. After testing clustering solutions using between 3 and 10 clusters, measures of cluster quality indicated that a five-cluster solution was best for both early-life and midlife trajectories (see the Supplementary Appendix for more detail). For both early-life and midlife we found a first cluster where respondents predominantly lived in big cities, a second where they predominantly lived in the suburbs, a third where they predominantly lived in large towns, a fourth where they mainly lived in small towns, and a final cluster where respondents principally lived in rural areas. Figure 1 shows the sequence density plots for the clusters for early-life and midlife residential trajectories.

We assessed cognitive reserve with two frequently used markers: education and the skill level of the respondents’ main job. Education was measured using the 1997 version of the International Standard Classification of Education (ISCED). We treated it as a categorical variable ranging from 0, no completed education, to 6, second-stage tertiary education. We used respondents’ highest reported level of education across all waves. Respondents were asked this question once and only reported any additional qualifications during follow-up. Over 99% of our sample reported no changes.

Job skill level was categorised in accordance with the four categories of skill level in the International Standard Classification of Occupations (ISCO) using the single-digit ISCO code of respondents’ main job over the life course. For respondents who were retired, but for whom data was missing, we used the ISCO code for the job they worked at the longest. From this we created a three-category variable distinguishing between high-skill occupations (skill levels 3 and 4 which are grouped together by the ISCO), low-skill occupations (skill levels 1 and 2)^28^, and respondents who never had any paid employment.

### Covariates

Our control variables included respondents’ age, sex, country fixed effects, and the individual means of self-rated health (SRH) and EURO-D depression scores across waves, two health status covariates known to be associated with cognitive function^29,30^. We centred age at 70 (the sample median) and divided it by 10 to prevent scaling issues and ensure convergence during estimation. We also included binary variables indicating whether respondents ever smoked and whether they consumed alcoholic beverages at least three to four times a week in the months before any survey interview, as these behavioural variables are known to influence cognitive function^31^. Descriptive statistics for the independent variables and covariates are shown in Table 4.

**Table 4:** Descriptive statistics for covariates.

**Early-life residency**	
Big city	5577 (14.6%)
Suburbs	2103 (5.5%)
Large town	5271 (13.8%)
Small town	7396 (19.4%)
Rural	17,818 (46.7%)
**Midlife residency**	
Big city	6761 (17.7%)
Suburbs	3339 (8.7%)
Large town	6147 (16.1%)
Small town	8549 (22.4%)
Rural	13,369 (35.0%)
**Residency during follow-up (number of observations)**	
Big city	18,435 (14.4%)
Suburbs	14,433 (11.3%)
Large town	21,163 (16.6%)
Small town	32,035 (25.1%)
Rural	41,401 (32.5%)
**Age**	69.7 (8.7)
**Sex**	
Male; female	15,789 (41.4%); 22,376 (58.6%)
**Job skill level**	
Never worked; low; high	3715 (9.7%); 24,195 (63.4%); 10,255 (26.9%)
**Education**	2.5 (1.5)
ISCED level 0	1861 (4.9%)
ISCED level 1	9296 (24.4%)
ISCED level 2	7006 (18.4%)
ISCED level 3	11,872 (31.1%)
ISCED level 4	1497 (3.9%);
ISCED level 5	6409 (16.8%)
ISCED level 6	224 (0.6%)
**Depressive symptoms (EURO-D)**	2.5 (1.9)
**Self-rated health (SRH)**	3.3 (0.9)
**Ever smoked**	
No; yes	20,672 (54.2%); 17,493 (45.8%)
**Frequent drinking**	
No; yes	24,956 (65.4%); 13,209 (34.6%)
**Country**	Austria: 2550 (6.7%); Belgium: 3195 (8.4%); Czech Republic: 3485 (9.1%); Denmark: 1591 (4.2%); Estonia: 2477 (6.5%); France: 2529 (6.6%); Germany: 2666 (7.0%); Greece: 2033 (5.3%); Hungary: 580 (1.5%); Ireland: 426 (1.1%); Israel: 898 (2.4%); Italy: 3222 (8.4%); Luxembourg: 472 (1.2%); Netherlands: 1323 (3.5%); Poland: 1468 (3.8%); Portugal: 624 (1.6%); Slovenia: 1580 (4.1%); Spain: 3333 (8.7%); Sweden: 2128 (5.6%); Switzerland: 1585 (4.2%)

Means and standard deviations (in parentheses) for continuous variables, number of cases and percentages (in parentheses) for categorical variables. Descriptives for time-varying variables (residency during follow-up and age) calculated using observations from verbal fluency models.

### Main statistical analysis

We estimated mixed-effects models using the lme4^32^ package with a random individual-specific intercept and a random slope for age for each cognitive functioning indicator. For each outcome, three models were estimated. The first included all control variables, country dummies, and residence during follow-up. The second model added early-life and midlife residential trajectories. The third model further included the two indicators of cognitive reserve (education and job skill level).

## Supplementary Information

Supplementary Information.

## Data Availability

This study uses data from Waves 1, 2, 3, 4, 5, 6, 7 and 8 from the Survey of Health, Ageing and Retirement in Europe (SHARE) (DOIs: 10.6103/SHARE.w1.710, 10.6103/SHARE.w2.710, 10.6103/SHARE.w3.710, 10.6103/SHARE.w4.710, 10.6103/SHARE.w5.710, 10.6103/SHARE.w6.710, 10.6103/SHARE.w7.711, 10.6103/SHARE.w8.100). The data that support the findings of this study are available from the Survey of Health, Ageing and Retirement in Europe but restrictions apply to the availability of these data, which were used under license for the current study, and so are not publicly available. Data are however available from the authors upon reasonable request and with permission of the Survey of Health, Ageing and Retirement in Europe. Researchers can register at http://www.share-project.org/data-access.html to obtain access to the data.

## References

[1] Hartley A, Angel L, Castel A (2018). Successful aging: The role of cognitive gerontology. Exp. Aging Res..

[2] Cassarino M, Setti A (2016). Complexity as key to designing cognitive-friendly environments for older people. Front. Psychol..

[3] Wu YT, Prina AM, Brayne C (2015). The association between community environment and cognitive function: A systematic review. Soc. Psychiatry Psychiatr. Epidemiol..

[4] Cerin E, Rainey-Smith SR, Ames D (2017). Associations of neighborhood environment with brain imaging outcomes in the Australian imaging, biomarkers and lifestyle cohort. Alzheimers Dement..

[5] Zhao G, Okoro CA, Hsia J (2019). Prevalence of disability and disability types by urban–rural county classification—U.S., 2016. Am. J. Prev. Med..

[6] Wu YT, Prina AM, Jones A (2017). The built environment and cognitive disorders: Results from the cognitive function and ageing study II. Am J Prev Med.

[7] Cassarino M, O'Sullivan V, Kenny RA, Setti A (2018). Disabilities moderate the association between neighbourhood urbanity and cognitive health: Results from the Irish longitudinal study on ageing. Disabil. Health J..

[8] Weden MM, Shih RA, Kabeto MU, Langa KM (2018). Secular trends in dementia and cognitive impairment of U.S. rural and urban older adults. Am. J. Prev. Med..

[9] Besser LM, McDonald NC, Song Y, Kukull WA, Rodriguez DA (2017). Neighborhood environment and cognition in older adults: A systematic review. Am. J. Prev. Med..

[10] Saenz JL, Downer B, Garcia MA, Wong R (2018). Cognition and context: Rural-urban differences in cognitive aging among older Mexican adults. J. Aging Health.

[11] Fritze T, Doblhammer G, van den Berg GJ (2014). Can individual conditions during childhood mediate or moderate the long-term cognitive effects of poor economic environments at birth?. Soc. Sci. Med..

[12] Herd P, Sicinski K, Asthana S (2021). Does rural living in early life increase the risk for reduced cognitive functioning in later life?. J. Alzheimers Dis..

[13] Wang XJ, Xu W, Li JQ, Cao XP, Tan L, Yu JT (2019). Early-life risk factors for dementia and cognitive impairment in later life: A systematic review and meta-analysis. J. Alzheimers Dis..

[14] Contador I, Bermejo-Pareja F, Puertas-Martin V, Benito-Leon J (2015). Childhood and adulthood rural residence increases the risk of dementia: NEDICES study. Curr. Alzheimer Res..

[15] Stern Y (2009). Cognitive reserve. Neuropsychologia.

[16] Lövdén M, Fratiglioni L, Glymour MM, Lindenberger U, Tucker-Drob EM (2020). Education and cognitive functioning across the life span. Psychol. Sci. Public Interest.

[17] Seblova D, Berggren R, Lövdén M (2020). Education and age-related decline in cognitive performance: Systematic review and meta-analysis of longitudinal cohort studies. Ageing Res. Rev..

[18] Lorenzo-López L, Millán-Calenti JC, López-López R (2017). Effects of degree of urbanization and lifetime longest-held occupation on cognitive impairment prevalence in an older Spanish population. Front. Psychol..

[19] Kuh D, Ben-Shlomo Y, Lynch J, Hallqvist J, Power C (2003). Life course epidemiology. J. Epidemiol. Community Health.

[20] Hirst RJ, Cassarino M, Kenny RA, Newell FN, Setti A (2021). Urban and rural environments differentially shape multisensory perception in ageing. Aging Neuropsychol. Cogn..

[21] Newbold KB, Brown WM (2015). The urban-rural gap in university attendance: Determinants of university participation among Canadian Youth. J. Reg. Sci..

[22] Brunello G, Weber G, Weiss CT (2017). Books are forever: Early life conditions, education and lifetime earnings in Europe. Econ. J..

[23] Robbins RN, Scott T, Joska JA, Gouse H (2019). Impact of urbanization on cognitive disorders. Curr. Opin. Psychiatry.

[24] Garrouste C, Paccagnella O, Schröder M (2011). Data quality: Three examples of consistency across SHARE and SHARELIFE data. Retrospective Data Collection in the Survey of Health, Ageing and Retirement in Europe: SHARELIFE Methodology.

[25] Börsch-Supan A, Brandt M, Hunkler C (2013). Data resource profile: The survey of health, ageing and retirement in Europe (SHARE). Int. J. Epidemiol..

[26] Gabadinho A, Ritschard G, Müller NS, Studer M (2011). Analyzing and visualizing state sequences in R with TraMineR. J. Stat. Softw..

[27] Studer M, Ritschard G (2016). What matters in differences between life trajectories: A comparative review of sequence dissimilarity measures. J. R. Stat. Soc. A. Stat. Soc..

[28] Ihle A, Gouveia ER, Gouveia BR (2018). The relation of hypertension to performance in immediate and delayed cued recall and working memory in old age: The role of cognitive reserve. J. Aging Health.

[29] Lee H, Schafer M (2021). Are positive childhood experiences linked to better cognitive functioning in later life?: Examining the role of life course pathways. J. Aging Health.

[30] Aichele S, Ghisletta P (2019). Memory deficits precede increases in depressive symptoms in later adulthood. J. Gerontol. B Psychol. Sci. Soc. Sci..

[31] Engelhardt H, Buber I, Skirbekk V, Prskawetz A (2010). Social involvement, behavioural risks and cognitive functioning among older people. Ageing Soc..

[32] Bates D, Mächler M, Bolker B, Walker S (2015). Fitting linear mixed-effects models Usinglme4. J. Stat. Softw..

