# Supplementary material for: Residential trajectories across the life course and their association with cognitive functioning in later life
Source: Sci Rep. 2022 Oct 11;12:17004. doi: 10.1038/s41598-022-18501-4 (PMC9553870; doi:10.1038/s41598-022-18501-4)
Supplement: Supplementary file 1 — Supplementary Information. [file 41598_2022_18501_MOESM1_ESM.pdf]

Residential Trajectories Across the Life Course and their Association with Cognitive Functioning in Later Life

Supplementary Material

Contents

- Assessment of Cognitive Functioning in SHARE
- Table S1: Pearson correlations between the different measures of cognitive functioning
- Measurement of Health and Depression in SHARE
- Sequence Analysis & Residential Trajectories
- Figure S1: Sequence Density Plots for Early-life Trajectories
- Figure S2: Sequence Density Plots for Midlife Trajectories
- Table S2: Cluster Quality Measures for Early-life Trajectories
- Table S3: Cluster Quality Measures for Midlife Trajectories

Assessment of Cognitive Functioning in SHARE
Immediate and delayed recall were assessed using a list of 10 words [1] read out by the interviewer. For the immediate recall score, respondents were asked to repeat as many words as they could remember right after they were read out to them. For delayed recall, respondents were asked to repeat the same list of 10 words after having responded to other questions assessing cognitive functioning. In the verbal fluency test [2], respondents were tasked with naming as many animals as possible in one minute. The score for this test was the total number of animals named that were validated by the interviewer, without any penalty for repetitions or wrong names. The Pearson correlations between the different outcomes are show in Table S1.

Table S1: Pearson correlations between the different measures of cognitive functioning.

	Immediate Recall	Delayed Recall	Verbal Fluency
Immediate Recall		0.720	0.488
Delayed Recall	0.720		0.458
Verbal Fluency	0.488	0.458	

Information on the Measurement of Self-rated Health and Depression in SHARE
SRH was treated as continuous scale ranging from 1 (Excellent) to 5 (Poor) and the EURO D scale [3] ranging from 0 (No depressive symptoms) to 12 (Highly depressed).

Sequence Analysis & Residential Trajectories
The residential trajectories were coded as sequences using the retrospective information supplied by respondents of the life-calendar, which is part of the SHARELIFE modules of waves three and seven. Respondents were asked about each place residence since their birth. They further provided information on when they began or stopped living at a certain place, and importantly, on the type of area a specific place of residence was in (big city, suburbs or peri-urban area, large town, small town, or rural area).

This information was imported in TraMineR [4] to analyse and classify the trajectories of respondents' area of residence. To cluster the trajectories, we calculated the distance between sequences using a spell-length optimized variant of optimal matching [5] with constant substitution costs. Because there were more than 40,000 sequences complete sequences for all respondents who completed the life history calendar, we used the WeightedCluster R package to select the unique sequences and calculate weights in relation to the number of times each unique sequence appeared in the original data. The clustering itself was done using Ward's method with the fastcluster R package using the calculated weights [6].

The choice of the number of groups was based on cluster quality measures computed with the WeightedCluster R package [7]. According to the cluster quality measures (Tables S2 and S3), the five clusters provided the best solution for midlife trajectories and is the best for early-life trajectories. Therefore, we partitioned the sequences into five clusters for both life phases. After a visual inspection of the sequences contained in each cluster (see Figures S1 and S2), we named the clusters as "big city" (cluster 1 for early-life trajectories and cluster 1 for midlife trajectories), "suburbs" (cluster 4 for early-life trajectories and cluster 3 for midlife trajectories), "large town" (cluster 3 for early-life trajectories and cluster 2 for midlife trajectories), "small town" (cluster 5 for early-life trajectories and cluster 4 for midlife trajectories), and "rural" residence (cluster 2 for early-life trajectories and cluster 5 for midlife trajectories) for both early-life and midlife trajectories. We then re-used the cluster assignments in the final analysis as an explanatory variable. The code for the analyses is available at: https://doi.org/10.17605/OSF.IO/TC8YE

Figure S1 Sequence density plots (chronograms) of early-life trajectories. For each cluster, the bars represent the proportion of respondents living in one of the five residential areas at a specific age in early life (birth to age 20). Based on this figure we see that cluster 1 mainly contains respondents who lived in a big city in early life, cluster 2 respondents who lived in rural areas, cluster 3 respondents who lived in large towns, cluster 4 respondents who lived in the suburbs, and cluster 5 respondents who lived in small towns.

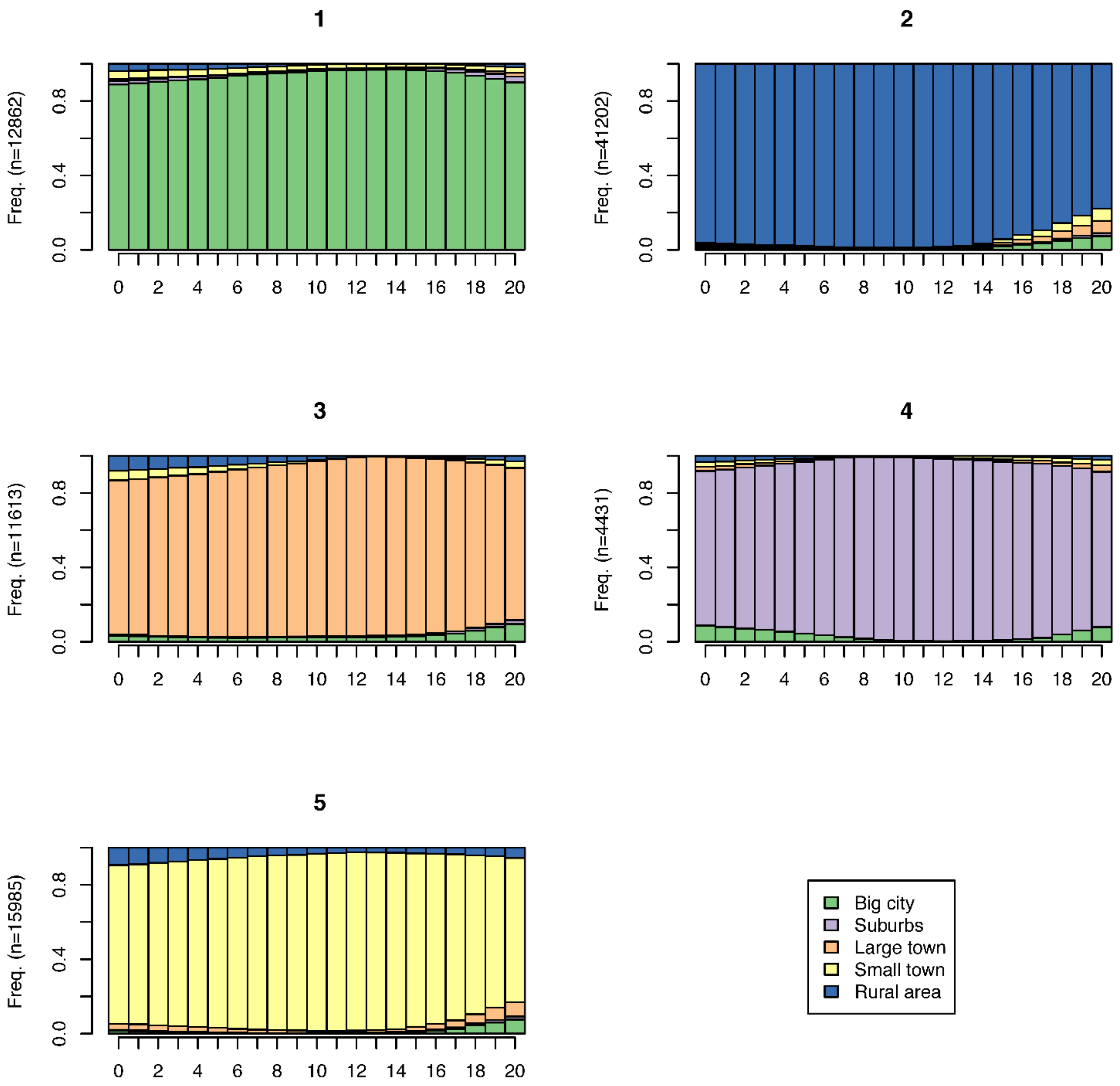

Figure S2 Sequence density plots (chronograms) of midlife trajectories. For each cluster, the bars represent the proportion of respondents living in one of the five residential areas at a specific age after early life (age 21 to study entry). Based on this figure we see that cluster 1 mainly contains respondents who lived in a big city, cluster 2 respondents who lived in large towns, cluster 3 respondents who lived in the suburbs, cluster 4 respondents who lived in small towns, and cluster 5 respondents who lived in rural areas.

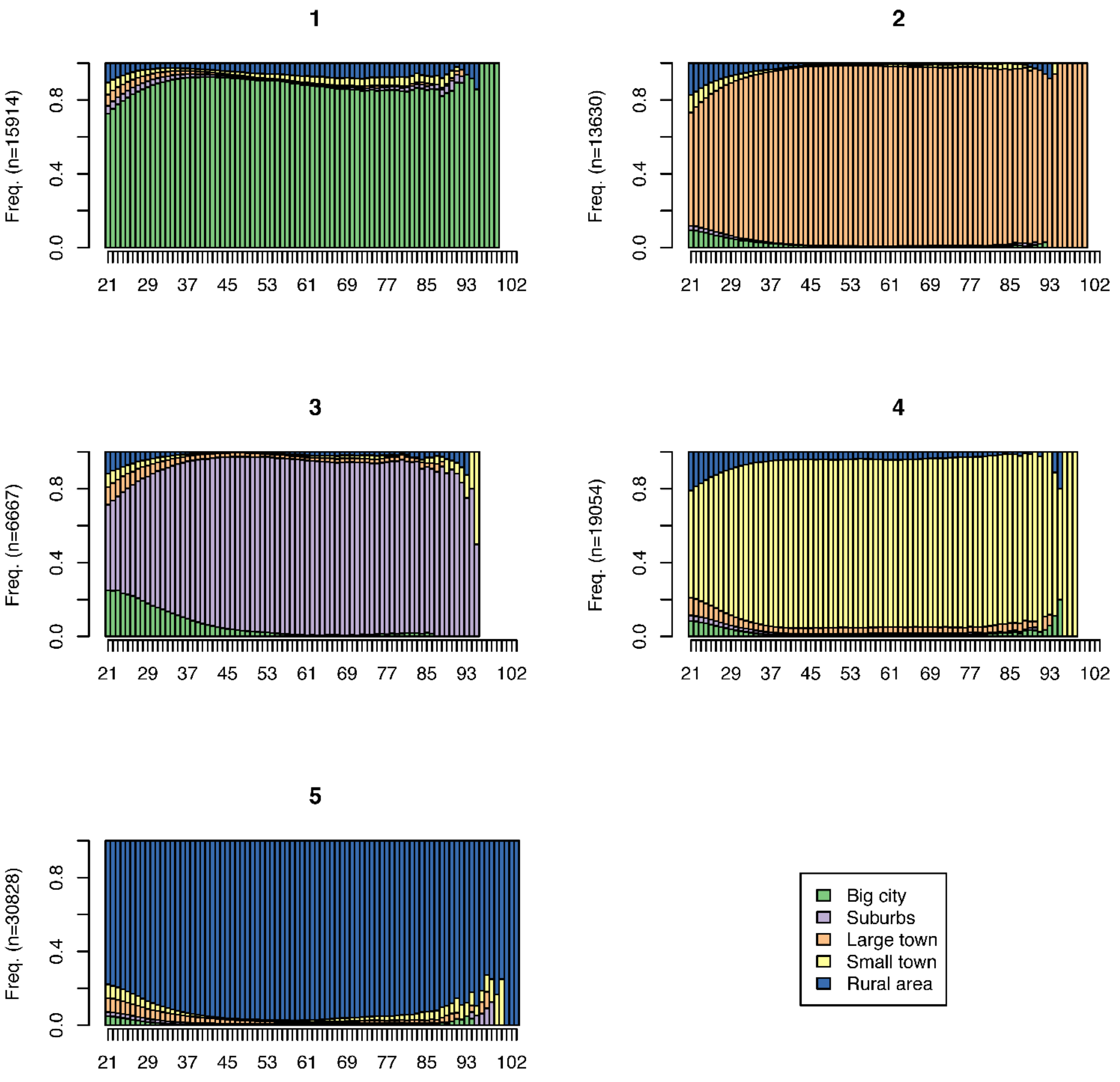

Table S2 Cluster quality measures for early-life sequences

For all measures but *HC*, a higher value indicates better clustering quality. The best clustering solution is the one with highest value for all measures other than *HC* and the one with the lowest HC value. PBC = Point Biserial Correlation, HG = Hubert's Gamma, HGSD = Hubert's Sommers' D, ASW = Average Silhouette Width, ASWw = Average Silhouette Width for weighted clusters, HC = Hubert's C.

**N Clusters**	**PBC**	**HG**	**HGSD**	**ASW**	**ASWw**	**HC**
**3**	0.803	0.849	0.825	0.656	0.656	0.071
**4**	0.909	0.963	0.956	0.756	0.757	0.030
**5**	**0.941**	**0.995**	**0.994**	**0.815**	**0.815**	0.010
**6**	0.941	0.995	0.994	0.782	0.782	0.010
**7**	0.941	0.995	0.994	0.777	0.777	0.010
**8**	0.939	0.994	0.994	0.769	0.769	0.011
**9**	0.940	0.995	0.995	0.759	0.759	0.010
**10**	0.940	0.995	0.995	0.756	0.756	**0.009**

Table S3 Cluster quality measures for midlife sequences

For all measures but *HC*, a higher value indicates better clustering quality. The best clustering solution is the one with highest value for all measures other than *HC* and the one with the lowest HC value. PBC = Point Biserial Correlation, HG = Hubert's Gamma, HGSD = Hubert's Sommers' D, ASW = Average Silhouette Width, ASWw = Average Silhouette Width for weighted clusters, HC = Hubert's C.

**N Clusters**	**PBC**	**HG**	**HGSD**	**ASW**	**ASWw**	**HC**
**3**	0.629	0.662	0.660	0.488	0.488	0.142
**4**	0.773	0.878	0.876	0.590	0.590	0.066
**5**	**0.825**	**0.966**	**0.965**	**0.646**	**0.646**	**0.030**
**6**	0.706	0.914	0.913	0.523	0.523	0.083
**7**	0.671	0.911	0.910	0.458	0.458	0.083
**8**	0.645	0.911	0.910	0.402	0.402	0.081
**9**	0.640	0.915	0.914	0.389	0.389	0.077
**10**	0.639	0.917	0.916	0.394	0.394	0.074

References

1. Harris SJ, Dowson JH. Recall of a 10-word list in the assessment of dementia in the elderly. *The British Journal of Psychiatry* 1982; **141**: 524-7.
2. Rosen WG. Verbal fluency in aging and dementia. *Journal of Clinical Neuropsychology* 1980; **2**(2): 135-46.
3. Prince MJ, Reischies F, Beekman AT, et al. Development of the EURO-D scale--a European, Union initiative to compare symptoms of depression in 14 European centres. *Br J Psychiatry* 1999; **174**: 330-8.
4. Gabadinho A, Ritschard G, Müller NS, Studer M. Analyzing and Visualizing State Sequences in R with TraMineR. *Journal of Statistical Software* 2011; **40**(4).
5. Studer M, Ritschard G. What matters in differences between life trajectories: a comparative review of sequence dissimilarity measures. *Journal of the Royal Statistical Society: Series A (Statistics in Society)* 2016; **179**(2): 481-511.
6. Müllner D. fastcluster: Fast Hierarchical, Agglomerative Clustering Routines for R and Python. *Journal of Statistical Software* 2013; **53**(9).
7. Studer M. WeightedCluster Library Manual: A Practical Guide to Creating Typologies of Trajectories in the Social Sciences with R. *LIVES Working Papers* 2013; **24**.
